# Long- and Short-Term Exposures to PM_10_ Can Shorten Telomere Length in Individuals Affected by Overweight and Obesity

**DOI:** 10.3390/life11080808

**Published:** 2021-08-10

**Authors:** Michele Carugno, Elisa Borroni, Luca Fedrizzi, Mirjam Hoxha, Luisella Vigna, Dario Consonni, Valentina Bollati, Angela Cecilia Pesatori

**Affiliations:** 1 Department of Clinical Sciences and Community Health, University of Milan, 20138 Milan, Italy; elisa.borroni@unimi.it (E.B.); luca.fedrizzi@guest.unimi.it (L.F.); mirjam.hoxha@unimi.it (M.H.); valentina.bollati@unimi.it (V.B.); angela.pesatori@unimi.it (A.C.P.); 2 Occupational Health Unit, Fondazione IRCCS Ca’ Granda Ospedale Maggiore Policlinico, 20138 Milan, Italy; luisella.vigna@policlinico.mi.it (L.V.); dario.consonni@policlinico.mi.it (D.C.)

**Keywords:** air pollution, particulate matter (PM), telomere length (TL), overweight, obesity, hypersusceptible

## Abstract

Reduced telomere length (TL) has been associated with increased risk of age-related diseases, most likely through oxidative stress and inflammation, which have also been claimed as mechanisms underlying health effects of air pollution exposure. We aimed to verify whether exposure to particulate matter with diameter ≤10 µm (PM_10_) affects TL. We recruited 1792 participants with overweight/obesity in Milan (Italy) in 2010–2015 who completed a structured questionnaire on sociodemographic data, gave a blood sample for TL measurement by real-time PCR, and were assigned air pollution and meteorological data of their residential address. In multivariate mixed-effects linear models (with a random intercept on PCR plate), we observed a −0.51% change in TL (95% confidence interval (CI): −0.98; −0.05)) per 10 µg/m^3^ increase in PM_10_ at the day of recruitment. A similar decreasing trend in TL was observed up to two weeks before withdrawal, with percentage changes as low as −1.53% (average exposure of the 12 days before recruitment). Mean annual exposure to PM_10_ was associated with −2.57% TL reduction (95%CI: −5.06; −0.08). By showing consistent associations between short- and long-term PM_10_ exposures and reduced TL, our findings shed light on the potential mechanisms responsible for the excess of age-related diseases associated with air pollution exposure.

## 1. Introduction

Telomeres are ribonucleoprotein complexes (repetitive nucleotide sequences TTAGGG) that cap the ends of chromosomes with the aim to prevent DNA degradation or end-to-end chromosome fusion [1,2,3]. Their length decreases with age and after each cellular division, as the DNA polymerase is unable to complete the replication of the third end of chromosomes [4]. In addition to cellular duplication, the rate of telomere shortening has been observed to be accelerated by exposure to environmental factors such as smoking, obesity, psychosocial stress, and certain dietary habits [5,6]. The genomic instability that follows telomere shortening is a well-established mechanism of cancer development [7], and some studies documented an increased risk of age-related disease (e.g., cancer, cardiovascular disease, diabetes, and Alzheimer’s disease) among individuals with reduced telomere length [8,9,10].

Reduced telomere length (TL) has also been associated with oxidative stress and systemic inflammation [11]. These phenomena have in turn been claimed as potential mechanisms underlying health effects of air pollution exposure, especially particulate matter (PM) [12,13]. PM is a mixture of solid and liquid particles that is found suspended in the air. Plenty of studies have documented its effects on human health, for both short- and long-term exposures [12], especially by showing associations with increased cardiovascular and respiratory mortality and morbidity, as well as with lung cancer [14,15,16,17]. The pulmonary and systemic inflammation and oxidative stress related to PM exposure may also influence TL, increasing the replication rate of cells and enhancing the extent of telomere loss during each replication [18]. Although an increasing number of studies examined the association between PM exposure and TL, results are still inconsistent, and their comparability is hampered by at least two factors: most studies involved quite a small number of participants; in addition, some studies investigated occupationally exposed individuals, such as truck drivers [19], steel workers [20], police traffic officers [21], and boilermakers [22]: In occupational settings, PM composition can be quite different (e.g., metal- or solvent-enriched) from environmental PM exposure.

Only a few studies evaluated the relationship between ambient air pollution and TL: McCracken and colleagues found a negative association between annual black carbon and TL in a sample of 165 non-smoking persons [23]; Pieters et al. found similar findings examining annual exposure to PM with diameter ≤2.5 µm (PM_2.5_) and TL in 166 Flemish elderly non-smokers [24].

Obesity is a well-established factor of telomere shortening, following induction of oxidative stress and acceleration of the rate of telomere erosion per replication [25]. In addition, obesity has been considered a susceptibility factor to the adverse effects of PM exposure, due to an increase in particle absorption and deposition in the lungs and to a higher low-grade chronic inflammation, which maximize the possible molecular response to air pollution [26].

The aim of the present study was to determine the effects of both short- and long-term exposures to PM with diameter ≤10 µm (PM_10_) on telomere length in a well-characterized population of individuals affected by overweight and obesity who were recruited within the SPHERE project (ERC-2011-StG 282413). The project was specifically designed to examine if PM exposure could modify microvesicles and microRNA content in the plasma of a large population of people with overweight/obesity; the project’s rationale and study protocol have been thoroughly described elsewhere [26]. Previous findings in subgroups of this population support the hypothesis of the hypersusceptibility of individuals with overweight/obesity to the effects of PM [27,28].

## 2. Results

In the present study, 1729 individuals affected by overweight and obesity were included. Seventy-three percent were females, and the mean age was 52.4 (SD: 13.7) years. Most of our study population had a secondary or high school degree, and more than 70% had a BMI ≥ 30. Among those for which information was available, about half reported no alcohol consumption or never smoking. Being male, having ≥61 years of age, and a lower level of education or a higher BMI, smoking ≥30 pack-years, living in a city or suburbs, and being affected by type 2 diabetes or metabolic syndrome were significantly associated with decreased telomere length in univariate analyses (Table 1).

**Figure 1:**
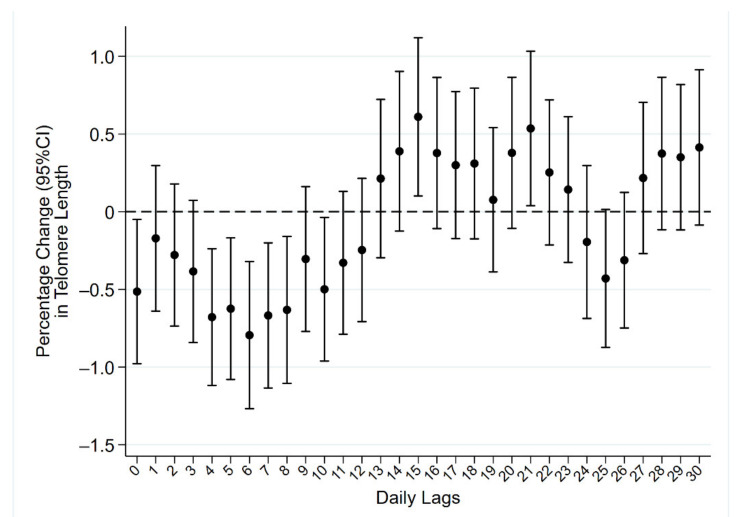
Short-term PM_10_ exposure and telomere length. Results expressed as percentage change in telomere length, with corresponding 95% confidence intervals (95%CI), per 10 μg/m^3^ increase in PM_10_ concentration occurring daily from the day of recruitment (lag 0) up to 30 days before (lag 30).

When we examined the association between daily PM_10_ exposure and TL in multivariate analyses (Figure 1), we observed a significant reduction in TL at the day of blood withdrawal (lag 0), with a percentage change of −0.51 (95%CI: −0.98; −0.05, *p* = 0.030; Table S1) per 10 µg/m^3^ increase in the pollutant concentration. A similar pattern was apparent considering exposures up to 12 days before recruitment (lag 12), with the highest reduction observed at lag 6 (−0.79, 95%CI: −1.27; −0.32, *p* = 0.001).

**Figure 2:**
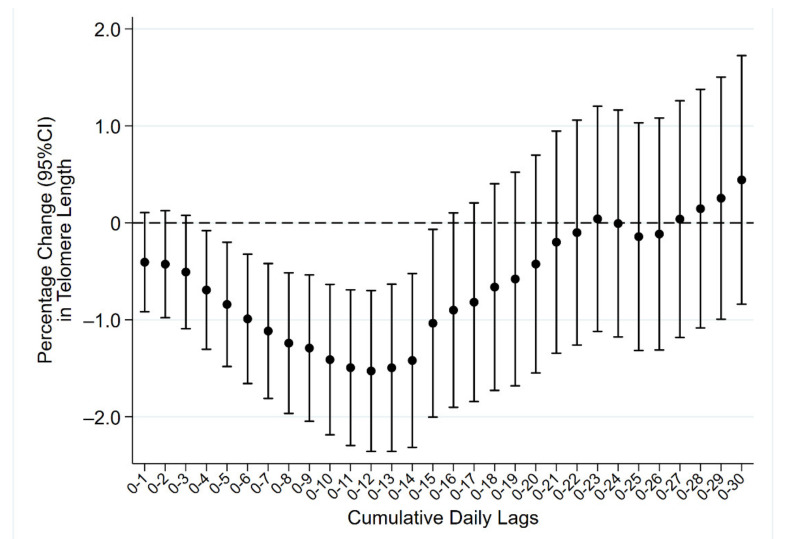
Short-term PM_10_ exposure and telomere length. Results expressed as percentage change in telomere length, with corresponding 95% confidence intervals (95%CI), per 10 μg/m^3^ increase in PM_10_ concentration values obtained by averaging PM_10_ levels of the day of recruitment with the levels of the day before (lag 0–1) and of each preceding day up to 30 days before (lag 0–30).

In cumulative daily lags analyses (i.e., considering the moving averages of exposure levels in the days preceding recruitment—Figure 2), we observed a decreasing trend in TL up to two weeks before withdrawal, with percentage changes ranging from −0.41 (lag 0–1) to −1.53 (lag 0–12). The effect of PM on TL gradually decreased while moving towards lag 0–30 (Table S2).

**Figure 3:**
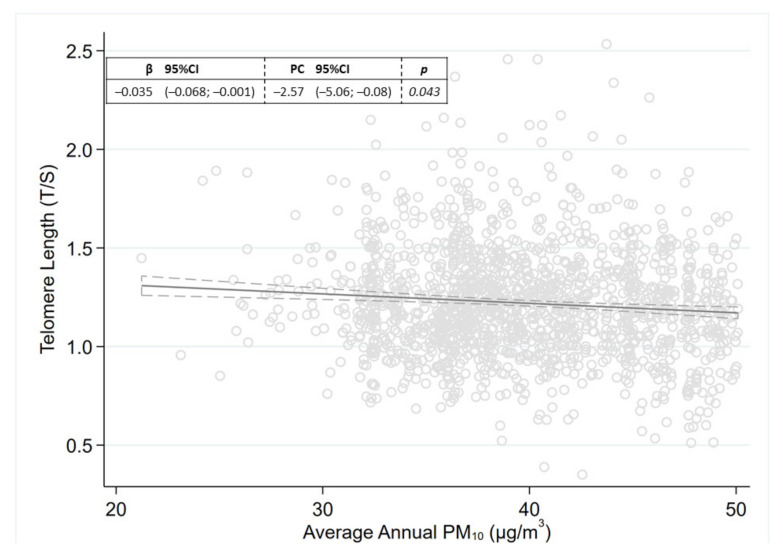
Long-term PM_10_ exposure and telomere length. Results expressed as regression coefficient (β) and percentage change (PC) in telomere length, with corresponding 95% confidence intervals (95%CI), per 10 μg/m^3^ increase in the average of PM_10_ levels of the 365 days preceding recruitment.

Long-term exposure to PM_10_ was associated with a −2.57% TL reduction (95%CI: −5.06; –0.08, *p* = 0.043) per a 10 µg/m^3^ increase in annual average PM_10_ levels (Figure 3). The direction of the association was comparable even when investigating the effect of PM on TL within categories of overweight/obesity, even if its magnitude seemed to decrease with increasing levels of BMI (Figures S1–S3).

The association between short-term exposure and TL remained practically unchanged when we additionally adjusted for long-term exposure (−1.08%, 95%CI: −1.94; −0.21, *p* = 0.015 at lag 0–14).

Further adjustment for metabolic syndrome and place of living did not substantially change our findings (data not shown). Analyses stratified by sex, age (≤60 vs. >60), BMI categories (<30 vs. 30–34.99 vs. ≥35), waist circumference (≤102 in males and ≤88 in females vs. >102 in males and >88 in females), and smoking status (current vs. former/never) did not show any effect modification. When stratifying by diabetes status (yes vs. no), results were substantially unaltered among non-diabetic participants, while associations disappeared when restricting our analyses to the 188 participants with diabetes.

## 3. Discussion

In a large population of persons affected by overweight and obesity, we found both short- and long-term air pollution exposures to be associated with reduced telomere length. The reduction was particularly evident in the two weeks before blood drawing and remained consistent even after taking into consideration the exposure of the preceding year. In addition, no interaction was found when considering several potential effect modifiers.

The only study examining the association between long-term exposure to environmental particulates and TL [24] was conducted in a population of nonsmoking elderly individuals and showed findings similar to ours (although of greater magnitude), with a 17% reduction in TL per each 5 μg/m^3^ increment in annual PM_2.5_ concentration. A non-significant association with decreasing TL was also found for annual cumulative PM_2.5_ exposure in a small sample of individuals occupationally exposed to welding fumes [22].

Although some studies have investigated the association between short-term air pollution exposure and TL [19,20,24,29], half of them were conducted in occupational settings, where PM composition can hardly be considered representative of environmental exposures. Only two studies addressed this research question considering environmental exposures but found inconsistent results. Xia and colleagues did not find an association between either PM_10_ or PM_2.5_ and TL measured in a very small sample of type 2 diabetes patients [29]. When we focused on subjects affected by diabetes, even our analyses showed no associations. On the other hand, Pieters et al. found an increase in TL for exposure to PM_2.5_ in the month prior to blood drawing. However, their study population consisted of individuals older than 60 years of age (while 70% of our participants were aged 60 or less), and no association was observed when considering the exposure of the week before recruitment [24]. Hence, heterogeneity in the reported associations could be explained by differences in the exposed populations (e.g., different age distributions or general vs. working populations), methods of exposure assessment, and PM composition in different settings (i.e., environmental vs. occupational).

Our study seems to return a more consistent picture, where both long- and short-term exposures to ambient particulates are able to influence telomeres by inducing their shortening.

The main mechanisms through which air pollution might be able to eventually damage telomeres by influencing their length are oxidative stress and inflammation [30]. In particular, reactive oxygen species are able to produce accumulation of single-strand nicks in telomeres, which are less easily repaired if compared with other genomic regions [31]. In addition, inflammation due to air pollution exposure might increase the replication speed of leukocytes, thus leading to accelerated shortening of TL [32].

Telomere length is also considered a marker of biological aging [33]. As such, the observed interlink between air pollution and decreased TL might partially explain the excess of age-related diseases, which is known to be associated with air pollution exposure [34].

The strength of our investigation mainly relies on its sample size, which is (to the best of our knowledge) the largest one among studies addressing the topic of air pollution and telomere length. We could rely on thorough control of individual characteristics, thus being able to minimize potential confounding. The contemporary analysis of both long- and short-term exposures allowed us to verify whether exposures in the days prior to blood drawing are able to affect telomeres, regardless of the chronic exposure of the study participants. Finally, we showed consistent results within a population of persons affected by overweight and obesity, which are known to be hypersusceptible to the chronic health effects related to air pollution exposure [35] and thus might contribute to unveil the biological mechanisms underlying this association.

Our study also has limitations. First, exposure assessment relies on air quality monitoring stations, whose measurements might inaccurately capture the real exposure at residential addresses. Second, we selected PM_10_ as the air pollutant of choice rather than PM_2.5_ because its data were more complete and had a better spatial resolution. Although PM_2.5_ represents a very high proportion of PM_10_ in the study area [36], this might also partly explain the different magnitude of the association observed by Pieters and colleagues [24]. Third, obesity is known as a condition characterized by a chronic low-grade inflammation. The alterations of adipocyte-derived signal mediators, in fact, strongly influence the regulation of inflammation [37]. Weight gain and obesity are also reported to promote telomere attrition regardless of age [38]. The findings obtained in the present investigation thus might be not completely applicable to the general population. Finally, we cannot exclude the possibility that telomere length might, at least in part, be affected by more chronic exposures (i.e., more distant in time than the year preceding participant recruitment).

## 4. Materials and Methods

### 4.1. Study Population

Recruitment criteria for the study population have been thoroughly described elsewhere [26]. Briefly, individuals with overweight/obesity were recruited at the Center for Obesity and Work (Fondazione IRCCS Ca’ Granda Ospedale Maggiore Policlinico in Milan, Lombardy, Italy) from September 2010 to March 2015. Study participants were eligible for inclusion if they met the following criteria: (1) being older than 18 years at the time of enrollment; (2) being affected by overweight (25 ≤ BMI < 30) or obesity (BMI ≥ 30); (3) being a resident in Lombardy at the time of the recruitment; and (4) agreeing to sign an informed consent and donate a blood sample. Participants were excluded when they had received a diagnosis of cancer, heart disease, or stroke in the last year or other chronic diseases such as multiple sclerosis, Alzheimer’s disease, Parkinson’s disease, depression, bipolar disorder, schizophrenia, and epilepsy.

### 4.2. Collection of Personal Data and Biological Samples

Each study participant was asked to complete a questionnaire including information on sociodemographic data (sex and age), residential area (home address, characteristics of the house, and traffic), education (i.e., primary school or less, high school, and university), smoking history (i.e., never, former, and current smokers; cigarettes smoked per day) including passive smoking at home and at workplace, alcohol habits (yes or no), past and present health status of both the participants and their first-degree relatives, physical activity levels and sedentary behavior, commuting time, and transport mode.

Each participant was asked to donate a 15 mL blood sample for biochemical and molecular tests. The blood sample was donated by venous phlebotomy after overnight fast and was collected into EDTA tubes (7 mL).

Methods to measure TL by real-time PCR were described elsewhere [39,40,41]. In brief, TL was measured by determining the ratio of telomeric repeat copy number (T) to a nuclear single copy gene (S, human beta-globin gene, HBG) copy number (T/S ratio) in a given sample relative to a reference pooled DNA used to generate a standard curve, which was inserted in each PCR run. Primer sequences have been reported elsewhere [39]. The reference pool DNA was prepared from 50 randomly selected DNA samples (4 µg DNA for each sample). A fresh 8-point standard curve prepared from the pooled DNA, ranging from 50 ng/µL to 0.39 ng/µL (serial dilutions 1:2), was included in every “T” and “S” PCR run. For each sample, 9 ng of DNA (concentration = 3 ng/ µL) was used as template and each reaction was run tripled. All PCR reactions were performed on a 7900HT Fast Real-Time PCR System (Applied Biosystems, Waltham, MA, USA) using 384-Well Block. At the end of each real-time PCR reaction, a melting curve was added in order to confirm the amplification specificity and the absence of primer dimers. The average of the three T measurements was divided by the average of the three S measurements to calculate the T/S ratio for each sample.

### 4.3. Exposure Assessment

Exposure to PM_10_ was evaluated by collecting daily concentrations from fixed monitoring stations of the Air Quality Monitoring Network of the Regional Environmental Protection Agency (ARPA Lombardia). From ARPA we also collected meteorological data, including temperature (233 monitors) and relative humidity (163 monitors). We used ArcGIS software (Esri) to assign to each participant the data measured from the monitoring station nearest to his/her home address. We calculated daily apparent temperature as a summary meteorological variable [42].

We evaluated both short- and long-term exposures. Short-term exposure to PM_10_ was assessed in different time windows, defined as (i) daily lags, obtained considering PM_10_ daily means from the day of recruitment (lag 0) up to 30 days before (lag 30); (ii) cumulative daily lags, obtained by averaging PM_10_ levels of the day of recruitment with the levels of the day before (lag 0–1) and of each preceding day up to 30 days before (lag 0–30). Long-term exposure was defined as the average of PM_10_ levels of the 365 days preceding the day of recruitment.

### 4.4. Statistical Analyses

After verifying the assumption of normality of our main dependent variable of interest (i.e., telomere length), we used standard descriptive statistics (i.e., means, standard deviations (SD), and proportions) to summarize data. We assessed the differences in the distribution of TL across different categories of selected variables using the chi-squared test, with a random intercept on the PCR plate (to properly take into account inter-plate variability).

Multivariate mixed-effects linear models with a random intercept on plate were run to evaluate the association between PM_10_ exposure and TL. Each model was adjusted for age, sex, BMI, level of education, time since quitting smoking, pack-years of smoking, and percentage level of neutrophils. Short-term exposure models were additionally adjusted for daily apparent temperature, year of recruitment, and season. Sensitivity analyses were conducted, adding metabolic syndrome and place of living as further adjusting covariates and stratifying for selected variables of interest to verify the presence of effect modification.

In order to evaluate the influence of long-term PM_10_ concentrations on short-term exposure, we built a multiple exposure model where we included PM_10_ annual average and lag 0–14 levels at the same time, adjusting for the same covariates, as previously specified.

Results are expressed as regression coefficients or slopes (β) and percentage change (PC) in TL per 10 µg/m^3^ increase in PM_10_ concentrations, with corresponding 95% confidence intervals (95%CI). Analyses were performed using Stata (Stata Corp. 2019; Stata Statistical Software: Release 16; College Station, Texas, USA: Stata Corp LLC).

## 5. Conclusions

In conclusion, our study consistently showed that both long- and short-term air pollution exposures shorten telomere length in a population of hypersusceptible individuals. Our findings thus shed light on the potential mechanisms responsible for the excess of age-related diseases that is known to be associated with air pollution exposure.

## Figures and Tables

**Table 1:** Descriptive statistics of the study population and distribution of telomere length (TL) across its main characteristics.

Characteristic	N (*%*)	TL Mean ± SD	p *
**Sex**			
Females	1259 (*72.8*)	1.04 ± 0.30	
Males	470 (*27.2*)	0.98 ± 0.28	*<0.001*
**Age**			
18–45	527 (*30.5*)	1.14 ± 0.30	
46–60	704 (*40.7*)	1.02 ± 0.28	
61+	498 (*28.8*)	0.92 ± 0.26	*<0.001*
**Education**			
None or primary school degree	141 (*8.2*)	0.88 ± 0.25	
Secondary or high school degree	1278 (*73.9*)	1.03 ± 0.29	
University degree or higher	281 (*16.2*)	1.09 ± 0.31	*<0.001*
Missing	29 (*1.7*)		
**BMI (3 cat)**			
Overweight (<30)	458 (*26.8*)	1.06 ± 0.32	
Obesity class I (30–34.99)	651 (*38.4*)	1.03 ± 0.29	
Obesity class II and III (≥35)	580 (*34.6*)	1.00 ± 0.27	*0.001*
Missing	3 (*0.2*)		
**Alcohol consumption**			
No	549 (*31.7*)	1.04 ± 0.28	
Yes	648 (*37.5*)	1.03 ± 0.30	*0.738*
Missing	532 (*30.8*)		
**Smoking status**			
Never	845 (*48.9*)	1.03 ± 0.29	
Former	611 (*35.3*)	0.99 ± 0.29	
Current	270 (*15.6*)	1.08 ± 0.30	*<0.001*
Missing	3 (*0.2*)		
**Pack-years of smoking**			
0	845 (*48.9*)	1.03 ± 0.29	
1–19	475 (*27.5*)	1.08 ± 0.30	
20–29	132 (*7.6*)	0.98 ± 0.26	
≥30	195 (*11.3*)	0.93 ± 0.27	*<0.001*
Missing	82 (*4.7*)		
**Place of living**			
City/Suburbs	1230 (*71.2*)	1.02 ± 0.29	
Rural area/Town	341 (*19.7*)	1.06 ± 0.29	*0.024*
Missing	158 (*9.1*)		
**Type 2 diabetes**			
No	1487 (*86.0*)	1.02 ± 0.30	
Yes	188 (*10.9*)	0.92 ± 0.26	*<0.001*
Missing	54 (*3.1*)		
**Metabolic Syndrome**			
No	927 (*53.6*)	1.07 ± 0.30	
Yes	766 (*44.3*)	0.98 ± 0.27	*<0.001*
Missing	36 (*2.1*)		
**Cancer**			
No	1656 (*95.8*)	1.03 ± 0.29	
Yes	73 (*4.2*)	0.99 ± 0.30	*0.168*
**TOTAL**	1729 (*100*)	1.03 ± 0.29	*-----*

* Chi-squared test, with a random intercept on PCR plate.

## Data Availability

Data are available upon reasonable request to the corresponding author.

## Supplementary Materials

The following are available online at https://www.mdpi.com/article/10.3390/life11080808/s1, Table S1: Short-term PM_10_ exposure and telomere length. Results expressed as regression coefficients (β) and percentage change (PC) in telomere length, with corresponding 95% confidence intervals (95%CI), per 10 μg/m^3^ increase in PM_10_ concentration occurring daily from the day of recruitment (lag 0) up to 30 days before (lag 30), Table S2: Short-term PM_10_ exposure and telomere length. Results expressed as regression coefficients (β) and percentage change (PC) in telomere length, with corresponding 95% confidence intervals (95%CI), per 10 μg/m^3^ increase in PM_10_ concentration values obtained by averaging PM_10_ levels of the day of recruitment with the levels of the day before (lag 0–1) and of each preceding day up to 30 days before (lag 0–30), Figure S1: Long-term PM_10_ exposure and telomere length among subjects with overweight (BMI < 30, number of non-missing observations = 415). Results expressed as regression coefficient (β) and percentage change (PC) in telomere length, with corresponding 95% confidence intervals (95%CI), per 10 μg/m^3^ increase in the average of PM_10_ levels of the 365 days preceding recruitment, Figure S2: Long-term PM_10_ exposure and telomere length among subjects with obesity class I (BMI = 30–34.99, number of non-missing observations = 592). Results expressed as regression coefficient (β) and percentage change (PC) in telomere length, with corresponding 95% confidence intervals (95%CI), per 10 μg/m^3^ increase in the average of PM_10_ levels of the 365 days preceding recruitment, Figure S3: Long-term PM_10_ exposure and telomere length among subjects with obesity class II and III (BMI ≥ 35, number of non-missing observations = 525). Results expressed as regression coefficient (β) and percentage change (PC) in telomere length, with corresponding 95% confidence intervals (95%CI), per 10 μg/m^3^ increase in the average of PM_10_ levels of the 365 days preceding recruitment.
