# Supplementary material for: Long- and Short-Term Exposures to PM10 Can Shorten Telomere Length in Individuals Affected by Overweight and Obesity
Source: Life (Basel). 2021 Aug 10;11(8):808. doi: 10.3390/life11080808 (PMC8400348; doi:10.3390/life11080808)
Supplement: Supplementary file 1 [file life-11-00808-s001.zip › life-1340055-supplementary.pdf]

Table S1. Short-term PM_{10} exposure and telomere length. Results expressed as regression coefficients (β) and percentage change (PC) in telomere length, with corresponding 95% confidence intervals (95%CI), per 10 μg/m^3 increase in PM_{10} concentration occurring daily from the day of recruitment (lag 0) up to 30 days before (lag 30).

Daily lags	β	95%CI	PC	95%CI	*p*
Lag 0	–0.006	(–0.012; –0.001)	–0.51	(–0.98; –0.05)	*0.030*
Lag 1	–0.002	(–0.008; 0.004)	–0.17	(–0.64; 0.30)	*0.474*
Lag 2	–0.003	(–0.009; 0.002)	–0.28	(–0.74; 0.18)	*0.232*
Lag 3	–0.005	(–0.010; 0.001)	–0.38	(–0.84; 0.07)	*0.100*
Lag 4	–0.009	(–0.014; –0.003)	–0.68	(–1.12; –0.24)	*0.002*
Lag 5	–0.008	(–0.013; –0.002)	–0.62	(–1.08; –0.17)	*0.007*
Lag 6	–0.010	(–0.016; –0.004)	–0.79	(–1.27; –0.32)	*0.001*
Lag 7	–0.008	(–0.014; –0.003)	–0.67	(–1.13; –0.20)	*0.005*
Lag 8	–0.008	(–0.014; –0.002)	–0.63	(–1.10; –0.16)	*0.009*
Lag 9	–0.004	(–0.010; 0.002)	–0.30	(–0.77; 0.16)	*0.200*
Lag 10	–0.006	(–0.012; –0.001)	–0.50	(–0.96; –0.04)	*0.034*
Lag 11	–0.004	(–0.010; 0.002)	–0.33	(–0.79; 0.13)	*0.161*
Lag 12	–0.003	(–0.009; 0.003)	–0.25	(–0.71; 0.21)	*0.295*
Lag 13	0.003	(–0.004; 0.009)	0.21	(–0.30; 0.72)	*0.412*
Lag 14	0.005	(–0.002; 0.011)	0.39	(–0.13; 0.90)	*0.138*
Lag 15	0.007	(0.001; 0.014)	0.61	(0.10; 1.12)	*0.019*
Lag 16	0.005	(–0.001; 0.011)	0.38	(–0.11; 0.86)	*0.128*
Lag 17	0.004	(–0.002; 0.010)	0.30	(–0.17; 0.77)	*0.214*
Lag 18	0.004	(–0.002; 0.010)	0.31	(–0.17; 0.79)	*0.210*
Lag 19	0.001	(–0.005; 0.007)	0.08	(–0.39; 0.54)	*0.747*
Lag 20	0.005	(–0.001; 0.011)	0.38	(–0.11; 0.86)	*0.127*
Lag 21	0.007	(0.001; 0.013)	0.54	(0.04; 1.03)	*0.035*
Lag 22	0.003	(–0.003; 0.009)	0.25	(–0.21; 0.72)	*0.289*
Lag 23	0.002	(–0.004; 0.008)	0.14	(–0.33; 0.61)	*0.552*
Lag 24	–0.002	(–0.009; 0.004)	–0.19	(–0.69; 0.30)	*0.437*
Lag 25	–0.006	(–0.011; 0.000)	–0.43	(–0.87; 0.01)	*0.058*
Lag 26	–0.004	(–0.010; 0.002)	–0.31	(–0.75; 0.12)	*0.160*
Lag 27	0.003	(–0.003; 0.009)	0.22	(–0.27; 0.70)	*0.382*
Lag 28	0.005	(–0.001; 0.011)	0.37	(–0.12; 0.86)	*0.135*
Lag 29	0.004	(–0.001; 0.010)	0.35	(–0.12; 0.82)	*0.142*
Lag 30	0.005	(–0.001; 0.011)	0.41	(–0.09; 0.91)	*0.105*

Table S2. Short-term PM_{10} exposure and telomere length. Results expressed as regression coefficients (β) and percentage change (PC) in telomere length, with corresponding 95% confidence intervals (95%CI), per 10 μg/m^3 increase in PM_{10} concentration values obtained by averaging PM_{10} levels of the day of recruitment with the levels of the day before (lag 0–1) and of each preceding day up to 30 days before (lag 0–30).

Cumulative daily lags	β	95%CI	PC	95%CI	*p*
Lag 0–1	–0.005	(–0.011; 0.001)	–0.41	(–0.92; 0.11)	*0.120*
Lag 0–2	–0.005	(–0.012; 0.002)	–0.43	(–0.98; 0.13)	*0.130*
Lag 0–3	–0.006	(–0.014; 0.001)	–0.51	(–1.09; 0.08)	*0.089*
Lag 0–4	–0.009	(–0.016; –0.001)	–0.69	(–1.30; –0.08)	*0.027*
Lag 0–5	–0.011	(–0.019; –0.003)	–0.84	(–1.48; –0.20)	*0.010*
Lag 0–6	–0.013	(–0.021; –0.004)	–0.99	(–1.66; –0.32)	*0.004*
Lag 0–7	–0.014	(–0.023; –0.005)	–1.12	(–1.81; –0.42)	*0.002*
Lag 0–8	–0.016	(–0.025; –0.007)	–1.24	(–1.97; –0.52)	*0.001*
Lag 0–9	–0.017	(–0.026; –0.007)	–1.29	(–2.05; –0.54)	*0.001*
Lag 0–10	–0.018	(–0.028; –0.008)	–1.41	(–2.19; –0.64)	*0.000*
Lag 0–11	–0.019	(–0.030; –0.009)	–1.49	(–2.30; –0.69)	*0.000*
Lag 0–12	–0.020	(–0.030; –0.009)	–1.53	(–2.36; –0.70)	*0.000*
Lag 0–13	–0.019	(–0.030; –0.008)	–1.49	(–2.36; –0.63)	*0.001*
Lag 0–14	–0.018	(–0.030; –0.007)	–1.42	(–2.32; –0.52)	*0.002*
Lag 0–15	–0.013	(–0.026; –0.001)	–1.04	(–2.00; –0.07)	*0.036*
Lag 0–16	–0.012	(–0.025; 0.001)	–0.90	(–1.90; 0.10)	*0.078*
Lag 0–17	–0.011	(–0.024; 0.003)	–0.82	(–1.84; 0.21)	*0.117*
Lag 0–18	–0.008	(–0.022; 0.005)	–0.66	(–1.73; 0.40)	*0.223*
Lag 0–19	–0.007	(–0.021; 0.007)	–0.58	(–1.68; 0.52)	*0.303*
Lag 0–20	–0.005	(–0.020; 0.009)	–0.42	(–1.55; 0.70)	*0.458*
Lag 0–21	–0.003	(–0.017; 0.012)	–0.20	(–1.34; 0.95)	*0.733*
Lag 0–22	–0.001	(–0.016; 0.014)	–0.10	(–1.26; 1.06)	*0.865*
Lag 0–23	0.001	(–0.015; 0.016)	0.04	(–1.12; 1.20)	*0.944*
Lag 0–24	–0.000	(–0.015; 0.015)	–0.01	(–1.18; 1.16)	*0.991*
Lag 0–25	–0.002	(–0.017; 0.014)	–0.14	(–1.32; 1.03)	*0.812*
Lag 0–26	–0.002	(–0.017; 0.014)	–0.12	(–1.31; 1.08)	*0.850*
Lag 0–27	0.001	(–0.016; 0.017)	0.04	(–1.18; 1.26)	*0.950*
Lag 0–28	0.002	(–0.014; 0.018)	0.15	(–1.08; 1.38)	*0.816*
Lag 0–29	0.003	(–0.013; 0.020)	0.25	(–0.99; 1.50)	*0.690*
Lag 0–30	0.006	(–0.011; 0.022)	0.44	(–0.84; 1.72)	*0.498*

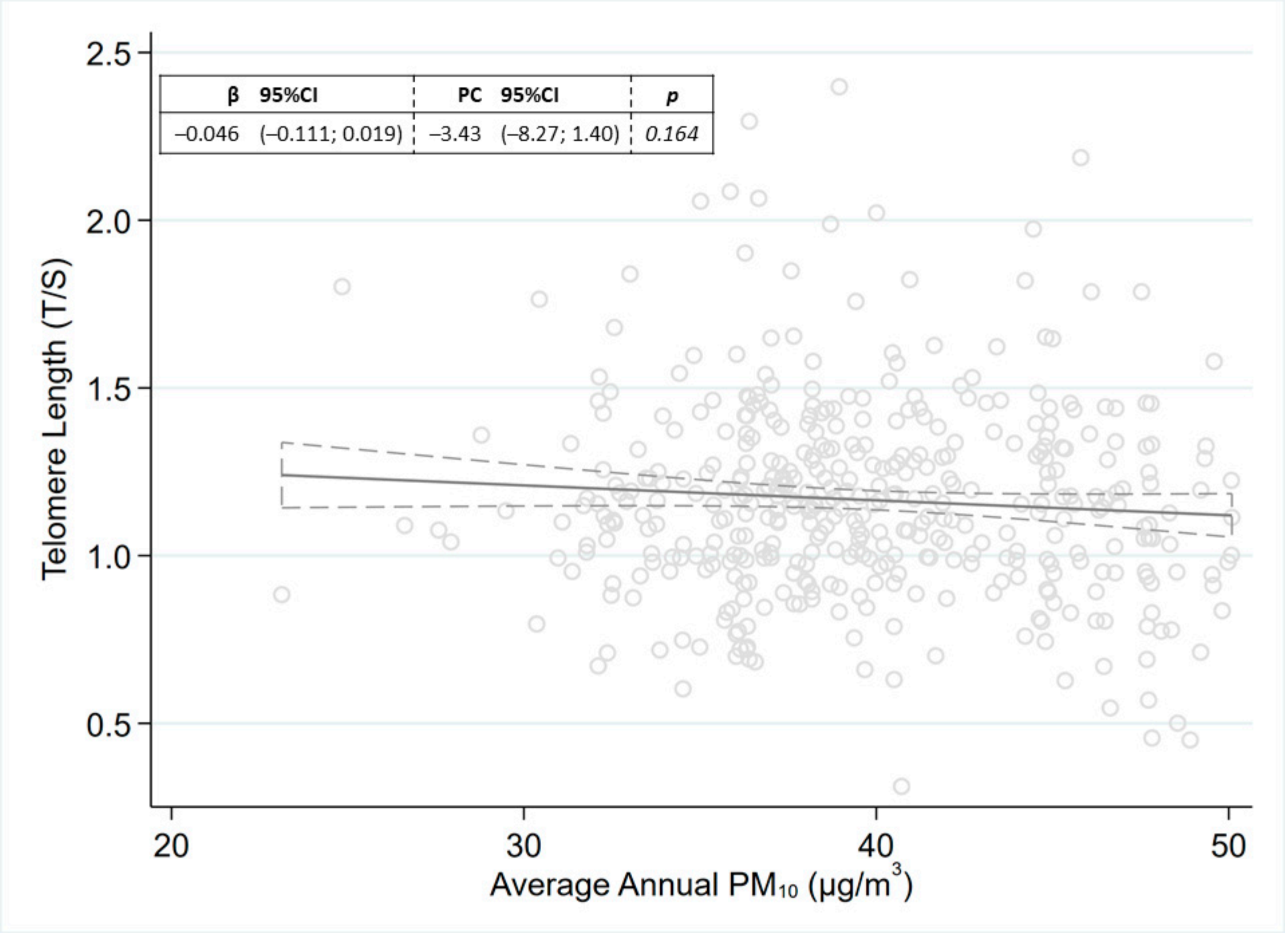

Figure S1. Long-term PM_{10} exposure and telomere length among subjects with overweight (BMI < 30, number of non-missing observations = 415). Results expressed as regression coefficient (β) and percentage change (PC) in telomere length, with corresponding 95% confidence intervals (95%CI), per 10 μg/m³ increase in the average of PM_{10} levels of the 365 days preceding recruitment.

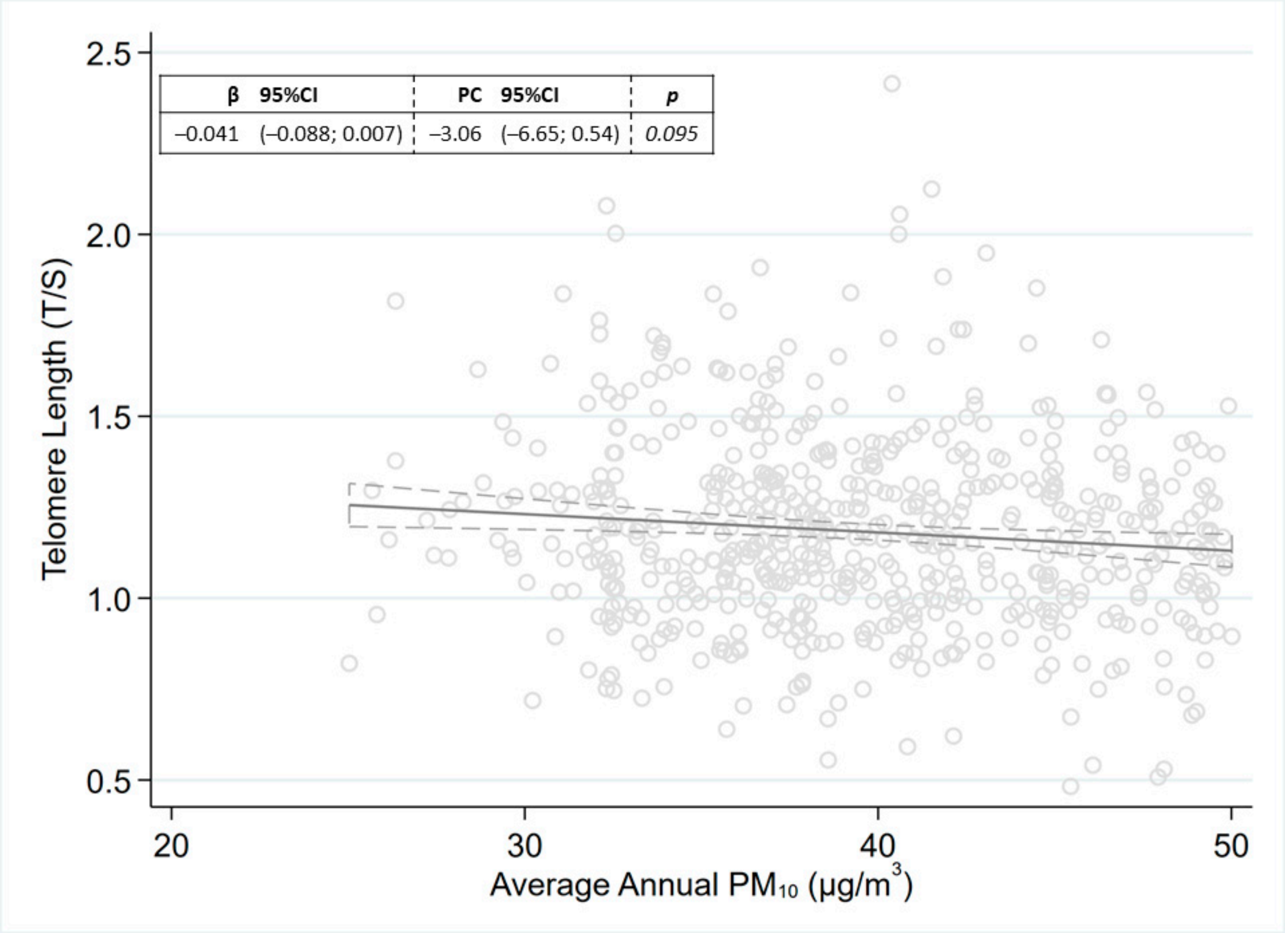

Figure S2. Long-term PM_{10} exposure and telomere length among subjects with obesity class I (BMI = 30–34.99, number of non-missing observations = 592). Results expressed as regression coefficient (β) and percentage change (PC) in telomere length, with corresponding 95% confidence intervals (95%CI), per 10 μg/m^3 increase in the average of PM_{10} levels of the 365 days preceding recruitment.

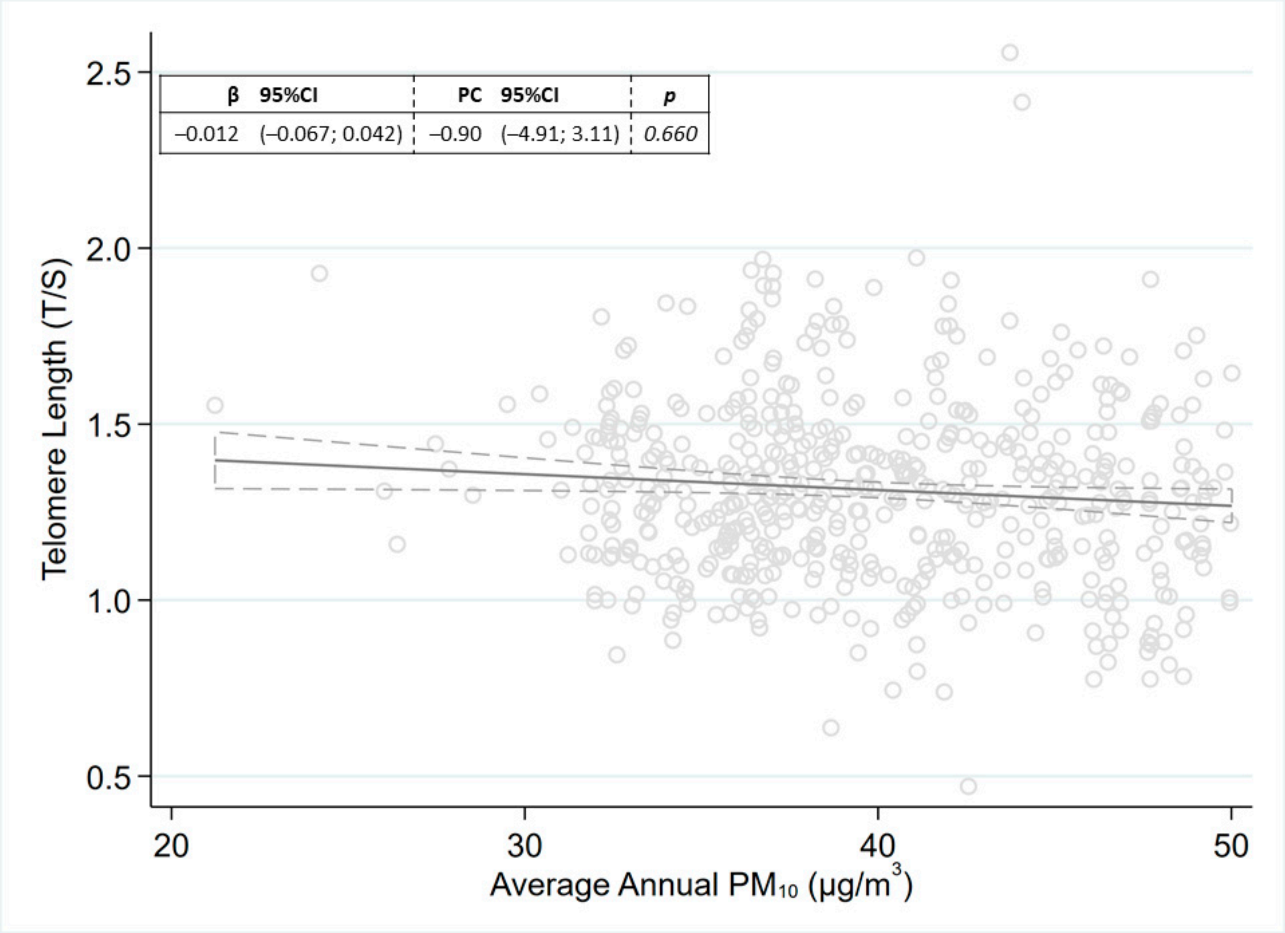

Figure S3. Long-term PM_{10} exposure and telomere length among subjects with obesity class II and III (BMI ≥ 35, number of non-missing observations = 525). Results expressed as regression coefficient (β) and percentage change (PC) in telomere length, with corresponding 95% confidence intervals (95%CI), per 10 μg/m^3 increase in the average of PM_{10} levels of the 365 days preceding recruitment.
